# Supplementary material for: Significance of serum FGF-23 for risk assessment of contrast-associated acute kidney injury and clinical outcomes in patients undergoing coronary angiography
Source: PLoS One. 2021 Jul 23;16(7):e0254835. doi: 10.1371/journal.pone.0254835 (PMC8301629; doi:10.1371/journal.pone.0254835)
Supplement: S1 Table — (DOCX) [file pone.0254835.s001.docx]

| **S1 Table. Baseline characteristics of patients with or without CA-AKI** | | | |
| --- | --- | --- | --- |
|  | With CA-AKI | Without CA-AKI |  |
|  | (n = 41) | (n = 451) | *P* |
| Age (years) | 71.5 ± 13.2 | 68.0 ± 12.3 | 0.523 |
| Male | 24 (58.5%) | 307 (68.1%) | 0.213 |
| Body mass index (kg/m2) | 24.7 ± 5.4 | 25.7 ± 4.2 | 0.271 |
| Current smoker | 11 (26.8%) | 152 (33.7%) | 0.371 |
| Hypertension | 33 (80.5%) | 292 (64.7%) | 0.042 |
| Diabetes mellitus | 20 (48.8%) | 145 (32.2%) | 0.031 |
| Chronic kidney disease | 24 (58.5%) | 124 (27.5%) | <0.001 |
| Prevalent HF and CVD |  |  |  |
| History of HF | 15 (36.6%) | 31 (6.9%) | <0.001 |
| History of MI | 2 (4.9%) | 25 (5.5%) | 1.000 |
| History of stroke/TIA | 1 (2.4%) | 25 (5.5%) | 0.713 |
| C-reactive protein (mg/dl) | 1.02 ± 1.07 | 0.55 ± 0.80 | 0.014 |
| eGFR (ml/min/1.73m^2^) | 57.0 ± 32.7 | 70.4 ± 21.2 | <0.001 |
| Contrast volume (ml) | 103 ± 68 | 99 ± 72 | 0.957 |
| Medication use |  |  |  |
| Loop diuretics | 14 (34.1%) | 34 (7.5%) | <0.001 |
| β-blocker | 10 (24.4%) | 88 (19.5%) | 0.454 |
| ACE inhibitor/ARB | 16 (39.0%) | 94 (20.8%) | 0.007 |
| Statin | 10 (24.4%) | 110 (24.4%) | 1.000 |
| Values are mean ± SD or number (%). | | | |
| CA-AKI: contrast-associated acute kidney injury; HF: heart failure; CVD: cardiovascular disease; MI: myocardial infarction; TIA: transient ischemic attack; eGFR: estimated glomerular filtration rate; ACE: angiotensin-converting enzyme; ARB: angiotensin receptor blocker | | | |
