# Supplementary material for: Significance of serum FGF-23 for risk assessment of contrast-associated acute kidney injury and clinical outcomes in patients undergoing coronary angiography
Source: PLoS One. 2021 Jul 23;16(7):e0254835. doi: 10.1371/journal.pone.0254835 (PMC8301629; doi:10.1371/journal.pone.0254835)
Supplement: Attachment — Submitted filename: Response to Reviewer 2.docx [file pone.0254835.s002.docx]

**Response to Comments by Reviewer #2**

Thank you very much for your interest in our paper and for your most instructive comments. We have revised the manuscript on the basis of your suggestions. The responses to your comments are as follow.

**Comments to the Author**

1. **The manuscript by Shao-Sung et al describes an interesting association between FGF-23 levels and CIN and MACE in patients submitted to coronary angiography. Despite the multivariate analysis, which included most comorbidites, findings are mainly observational and a causal relationship between FGF-23 and CIN and outcomes could not be demonstrated. Nevertheless, a significant association was clearly found. Accordingly, I suggest that in the causal relationship discussion, authors should include that FGF-23 is likely signaling a worse health status in the FGF-23 upper-level group, since this group presented significantly more comorbidities (DM, CKD, previous MI and stroke) and more importantly, higher inflammation (higher CRP) and especially lower eGFR. Therefore, it is very likely that this worse health status was actually responsible for the results: greater CIN and worse MACE. For this reason, I suggest that the statement, in both conclusions (abstract and main text), about reduction or modulation of FGF-23 could prevent CIN should be removed since a causal relationship was not proven. Instead, authors should include the MACE analysis together with CIN results in the conclusions.**

***Response:***

Thank you for your suggestions and recommendations. As you recommended, we have made the appropriate revisions in the manuscript.

1. **Abstract:**

**Elevated FGF-23 levels were associated with an increased risk for CA-AKI and future MACE in patients undergoing coronary angiography. FGF-23 may play a role in early diagnosis of CA-AKI and predicting clinical outcomes after coronary angiography. *(Please see page 3/line 1–4)***

2. **Discussion:**

In conclusion, FGF-23 is likely signaling a worse health status in the FGF-23 upper-level group, since this group presented significantly more comorbidities (DM, CKD, previous MI and stroke) and more importantly, higher inflammation (higher CRP) and especially lower eGFR. Therefore, it is very likely that this worse health status was actually responsible for the results: greater CA-AKI and worse MACE. Nevertheless, our results show promise for testing this biomarker as part of novel risk prediction models of renal and cardiovascular outcomes in patients undergoing coronary angiography. ***(Please see page 18/line 11–18)***

Note: Because using a more contemporary term such as contrast-associated acute kidney injury (CA-AKI) rather than contrast-induced nephropathy (CIN) was recommended by Reviewer #1, we have made the appropriate revision in the manuscript.

Contrast-induced nephropathy, also known as contrast-associated acute kidney injury (CA-AKI), remains a serious clinical problem associated with the use of iodinated contrast media in diagnostic imaging and interventional procedures. ***(Please see page 4/line 2–3)***

2. **Figures - level of significance should be pointed or demonstrated between which groups (upper vs. lower)**

***Response:***

Thank you for your suggestions and recommendations. As you recommended, we have made the appropriate revisions in the manuscript. ***(Please see Figure 1 & Figure 2)***

3. **Figure 1 - correct intermidiate title group (Intermediate)**

***Response:***

Thank you for your suggestions and recommendations. As you recommended, we have made the appropriate revisions in the manuscript. ***(Please see Figure 1)***

***Thank you very much again and we appreciate your comments!***
